# A bedridden young lady with hypophosphatemic rickets treated with denosumab: a case report

**DOI:** 10.1186/s13256-020-02654-9

**Published:** 2021-02-07

**Authors:** Butheinah A. Al-Sharafi, Nuha A. Al-Yousfi, Said A. Bamashmus

**Affiliations:** 1 Department of Medicine, School of Medicine and Health Sciences, Sana’a University, PO Box 12268, Sana’a, Yemen; 2 Department of Orthopedic Surgery, University of Science and Technology Hospital, PO Box 13061, Sana’a, Yemen

**Keywords:** Hypophosphatemic rickets, Denosumab, Osteomalacia, Hyperparathyroidism, Osteoporosis, Case report

## Abstract

**Background:**

Hypophosphatemic rickets is associated with delayed walking, bone deformities, growth failure and physical dysfunction that can limit daily activities. Treatment consists of phosphate salts and calcitriol. We report a case that received denosumab with marked improvement in her condition.

**Case presentation:**

A 24-year-old Yemeni female with hypophosphatemic rickets presented to an endocrinologist with severe weakness and severe pain in the extremities, she had been bedridden for the last 4 years. Bone density showed severe osteoporosis (*T* score of hip was − 5.0 and *Z* score of hip was − 5.0, *T* score of the spine was − 6.0 and *Z* score of the spine was − 6.1) so the patient was started on denosumab in addition to calcitriol and after 7months she was feeling stronger and felt she could stand assisted and was walking with assistance within 9 months and after 1.5 years of treatment she was walking unassisted.

**Conclusion:**

Denosumab is an effective treatment for osteoporosis, we used it in our patient in addition to calcitriol because she had severe osteoporosis due to long standing hypophosphatemic rickets that had not been treated properly, the patient improved markedly and regained the ability to walk again after being bedridden for 4 years. It may be a drug to consider in such cases although further studies need to be done to confirm this.

## Background

Hypophosphatemic rickets in children is revealed by delayed walking, waddling gait, leg bowing, enlarged cartilages, dental abscesses, bone pain, craniosynostosis and growth failure [1]. If undiagnosed in childhood it should be suspected if patients present with bone and/or joint pain, fractures, mineralization defects as osteomalacia, dental anomalies, hearing loss and fatigue [1, 2]. The disease may initially be misdiagnosed for vitamin D deficiency, but biochemical investigations and in some cases lack of response to vitamin D supplements usually allow the diagnosis [3]. The treatments available for these cases are phosphate and calcitriol till recently a study showed that burosumab could be used in these children with marked improvement [4]. We describe a case of hypophosphatemic rickets who was treated with cacitriol and then received denosumab for severe osteoporosis with marked improvement in her condition, and after being bedridden for 4 years she regained the ability to walk again without assistance.

## Case report

A 24-year-old Yemeni female presented to the endocrinology clinic in April 2017 because of inability to walk and a long history of osteomalacia. She was unable to walk for the last 4 years with severe weakness and bone pain. She reported that for the last 2 years she was so weak that she could not turn over in bed and this was associated with pain all over her body. Her condition started as a baby as she did not walk till she was 2.5 years old. Then when she started school she stated she could walk but could never run, the family was poor and never sought medical help for her condition. She got married at a young age of 15 and became pregnant soon after that. During her pregnancy she had difficulty in walking and had pain in her legs, she needed assistance to rise from the sitting position and she was told she would need a cesarean section because she had a contracted pelvis, but she went into labor before she was able to have a cesarean section and she delivered vaginally and developed a fracture of the pubic rami during delivery.

Her condition over the following years worsened and she was having more difficulty in walking and needed assistance in climbing stairs. She went to several orthopedic surgeons and she was diagnosed as osteomalacia and given calcium and vitamin D without any improvement, till she became totally bedridden. At one time she was also given calcitriol in small doses and for short periods of time 0.25 mg daily. She eventually stopped all treatments when she did not see any improvement in her condition. She was also seen by a neurologist and was found to have a normal nerve conduction study and was told there was no evidence of neurological disease.

She has no family history of a similar condition in her parents or siblings or other relatives.

At her initial presentation, physical examination of the patient showed that she was 144 cm tall and weighed 49 kg, she was much shorter than the rest of her family. She had normal teeth and hair development. Examination was within normal limits except that she had generalized weakness and tenderness in her extremities with bowing of the legs and genu valgus. All the blood work that the patient had from 2012 to 2019 can be seen in Table 1.

**Table 1:** The patients’ blood work from 2012 through 2019

Date	Ca (N 8.4–10.2 mg/dl	Mg (N 1.7–2.6 mg/dl)	Phos (N 2.3–4.7 mg/dl)	PTH (N 15–65 Pg/ml)	Alk phos (N 60–170 U/l)	Vit D (N 30–100 ng/ml)	1,25 OH Vit D (N 24–65 pg/ml)	TSH (N 0.27–4.2 uIU/ml)	HB (N 12–16 g/dl	24 hour urine ca (N 100–321 mg/24 hour)	24 hour urine phos (N 40–136 mg/24 hour)	Anti TG IgA (N negative)	Cr (N 0.6–1.1 mg/dl)
2/12	10.7								13.9				0.7
4/13	10.4	0.8			719	17.3							
5/13	10	0.8	2.1		401								
6/13	10.8	0.59	2.0		797								
3/15						38	13.5						
1/16				549		53		3.82	11.1				
4/17^a^	10.4			515								neg	0.5
7/17	9.4		1.4						15.5	678	294		
11/18	10.2	1.9	2.4	313						380			
11/19	12.8	1.7	2.7	239	238				13.9				0.67
11/19	12.2					120				407			0.37

*Ca* calcium, *Mg* magnesium, *PO*_*4*_ phosphorus, *PTH* intact parathyroid hormone, *Alk phos* alkaline phosphatase, *Vit D* Vitamin D, *1,25 OH Vit D* 1,25 dihydroxy vitamin D, *TSH* thyroid stimulating hormone, *Hb* hemoglobin, *24 hour urine ca* 24 hour urinary calcium excretion, *24 hour urine phos* 24 hour urinary phosphorous excretion, *anti TG IgA* anti transglutaminase IgA antibodies, *Cr* creatinine

^a^First presentation to our clinic.

## Investigations

**Fig. 1:**
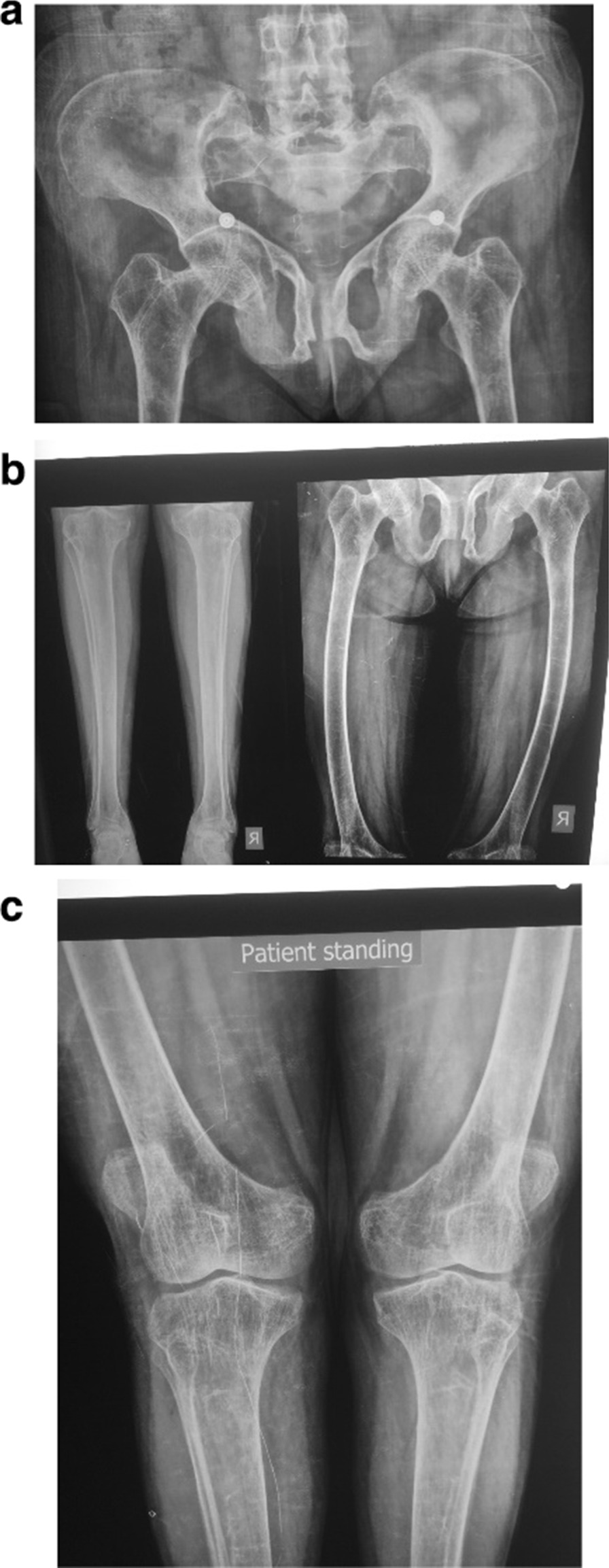
**a** Pelvic X-ray. Osteopenia around hip joints. Abnormal shaped pelvic brim and abnormal shaped pubic bones and acetabular fossa. Preserved joint spaces of both hips. **b** Leg X-rays. Bowing of both femurs, and mild osteopenia mainly around ankle joints. **c** Knee X-rays. Osteopenia mainly around knee joints. Bowing of both femurs and bilateral genu valgus deformity and prominent epicondyles

**Fig. 2:**
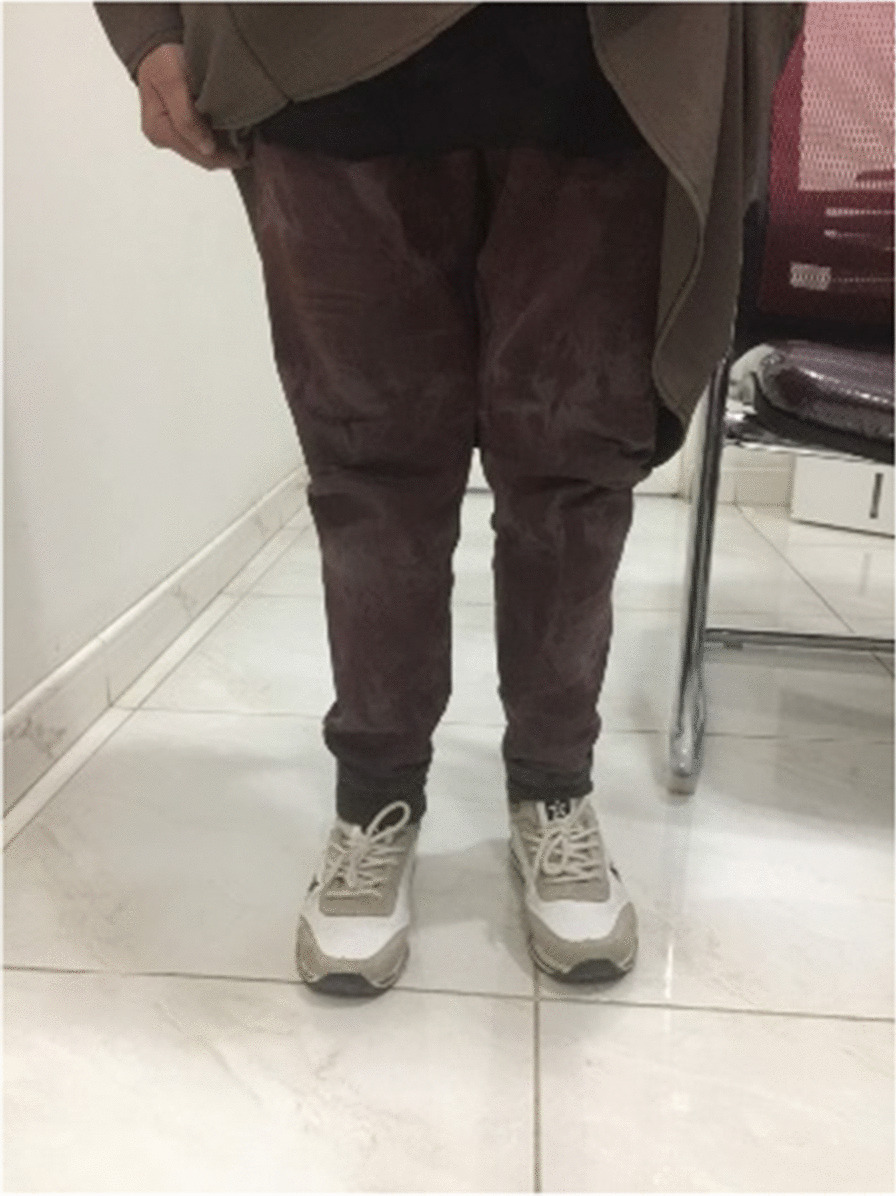
Picture of patient standing.

Bone density at presentation (April 2017) showed severe osteoporosis. Results of the bone density scans can be seen in Table 2. The case was reviewed with other doctors and she was diagnosed to have vitamin D resistant rickets from her history and clinical exam and lab results. She had a long history of rickets not responding to the usual forms of treatment, short stature the low 1,25 dihydroxyvitamin D level, in addition to the low phosphorous level, high alkaline phosphatase and X-ray findings. Fanconi syndrome is another differential diagnosis which can cause rickets, hypophosphatemia and growth failure but she did not have hypovolemia, hypokalemia or persistant acidosis and celiac disease was ruled out by checking the antitransglutamine IgA antibodies which were negative in addition to absence of gastrointestinal symptoms. She was started on calcitriol 1 mcg twice daily and calcium and vitamin D3 were also continued for a short period then tapered off. She was not given phosphate salts because it was not available in the country (due to the war and embargo many essential medications have not available in Yemen in these past years). Since the patient had severe osteoporosis she was started on denosumab 60 mg subcutanously every 6 months in June 2017. She gradually felt stronger on calcitriol and soon after her second dose of denosumab she stated that she could stand assisted by others, so the patient was sent for physiotherapy and within a couple of months she was able to walk with the aid of a walker, she continued to grow stronger and now can walk without assistance. She was receiving calcitriol 1 mcg daily till her most recent visit when her calcium was found to have increased but she denied having any symptoms of hypercalcemia. She had been receiving denosumab 60 mg subcutaneously every 6 months for 2 years. The patient had consistently high parathyroid hormone levels associated with a normal to high normal calcium level and a high 24-hour urine calcium level which is going with an associated primary hyperparathyroidism. Ultrasound of the neck did not show any parathyroid adenomas. Ultrasound of the kidneys was normal with no nephrocalcinosis. Sestamibi scan was not done due to inavailablity. Her most recent X-rays can be seen in Fig. 1a–c, also a picture of her standing can be seen in Fig. 2. At her last visit she was found to have an elevated Vitamin D3 level which might have been an additional factor contributing to the recent increase in the calcium level so calcitriol was discontinued temporarily till her next denosumab injection which is due in one month when she will be reassessed. The patient is now doing well, she is happy to be walking again and has no complaints.

**Table 2:** Bone density reports from April 2017-November 2019

Date	*T* score hip	*Z* score hip	*T* score spine	*Z* score spine
4/17	− 5.0	− 5.0	− 6.0	− 6.1
12/17	− 4.8	− 4.9	− 4.8	− 4.9
11/19	− 3.4	− 3.4	− 3.9	− 4.0

## Discussion

We describe a patient who had hypophosphatemic rickets who was bedridden by her disease because of severe weakness and generalized bony aches. At the time of her presentation she was not on any medications and was started on calcitriol and started showing improvement in her muscle power. The patient was found to have severe osteoporosis so denosumab was added 2 months later, the condition of the patient continued to improve. Her phosphorous level started to improve approximately 7 months after treatment which is probably the major factor in the improvement of her muscle weakness.

Denusumab is a human immunoglobulin G2 monoclonal antibody that inhibits bone resorption by targeting RANKL, which is involved in osteoclast differentiation. It has been used successfully to treat osteoporosis, lytic lesions associated with bony metastasis and diseases with osteoclast overactivity, including giant cell tumors of the bone. It has been used off label to treat other diseases of the bone with similar osteoclastic pathology including central giant cell granuloma, aneurysmal bone cysts and fibrous dysplasia [5]. The condition of the patient improved markedly on the medication. The patient did not develop any side effects from the denosumab such as hypocalcemia most likely due to the associated primary hyperparathyroidism in this patient. On reviewing the literature there were no cases reported on using denosumab for hypophosphatemic rickets but is has been used in a case of hypophosphatemic osteomalacia which was drug induced [6] it also has been reported to be used in cases of osteogenesis imperfecta [7]. Denosumab, in addition to other drugs are currently being investigated in phase III trials for use in hypophosphatemic rickets, hypophosphatasia and fibrodysplasia ossificans progressiva [8]. Our patient had hyperparathyroidism which is one of the complications when treating such patients with calcitriol and phosphate supplements [2, 3]. The level of alkaline phosphatase and parathyroid hormone levels although still high were decreasing over time. Calcitriol was discontinued at this time due to a high Vitamin D level and the recent increase in the calcium level. Nephrocalcinosis has been reported to occur in 34% of cases and tertiary hyperparathyroidism in 6% of patients in one study [3]. It is mostly reported to occur secondary to prolonged treatment with phosphate [9, 10]. We are not aware that our patient ever received phosphate salts in the past. Most likely our patient has primary hyperparathyroidism in addition to hypophosphatemic rickets. There has been a case reported to have hypophosphatemic rickets and primary hyperparathyroidism due to a de novo transloction with a breakpoint adjacent to α-Klotho, which encodes a β-glucoronidase, and is implemented in aging and regulation of FGF signaling [11]. We will continue to treat her with denosumab and if her calcium remains high she may need cinalcacet or parathyroidectomy.

## Conclusion

Our patient had hypophosphatemic rickets that was very advanced causing the patient to be bedridden. She received calcitriol and denosumab was added for severe osteoporosis and improved markedly and can now walk unassisted. Her improvement in muscle power was most likely due to calcitriol and normalization of the phosphate levels with denosumab helping in improvement of the bone strength. We did not anticipate this response to her treatment as she came in a very advanced stage being bedridden for 4 years. Denosumab may be a medication that could be considered in cases of hypophosphatemic rickets but further studies need to be done to prove its’ benefit in such cases.

## Data Availability

All the investigation results and reports are available on request
